# The Impact of Novel Lipid-Lowering Agents on Cardiovascular Risk Reduction: A Systematic Review and Meta-Analysis

**DOI:** 10.2174/011573403X345749250122092324

**Published:** 2025-02-11

**Authors:** Rubela Ray, Arhum Mahmood, Raheel Chaudhry, Mohd Diya Masmoum, Muhammad Talha, Fahad I. Siddiqui, Imdad Ullah

**Affiliations:** 1 Bankura Sammilani Medical College, Bankura, India;; 2 Henry Ford Health System, Detroit, MI 48202, USA;; 3 Baylor College of Medicine, Houston, TX 77030, USA;; 4 Alfaisal University, Riyadh, Saudi Arabia;; 5 Nishtar Medical University, Multan, Pakistan;; 6 Multan Medical and Dental College, Multan, Pakistan;; 7 Khyber Medical College, Peshawar, Pakistan

**Keywords:** PCSK9 inhibitors, lipid-lowering agents, atherosclerotic cardiovascular disease, ezetimibe, statin therapy, major adverse cardiovascular events, LDL cholesterol reduction, cardiovascular risk reduction

## Abstract

**Introduction:**

Reducing the risk of atherosclerotic cardiovascular disease is the aim of lipid-lowering therapy (ASCVD). It is commonly acknowledged that low-density lipoprotein (LDL) is a major cause of ASCVD. Several online databases and search engines, such as PubMed and the Cochrane Library, were used to conduct a thorough search.

**Methods:**

This study included RCTs assessing the effect of PCSK9 inhibitors on cardiovascular events. The RevMan 5.4 software was used to conduct the meta-analysis. This analysis included nine RCTs in total.

**Results:**

Meta-analysis of the included studies showed that the levels of total cholesterol, LDL, and triglycerides were reduced after the use of PCSK9 inhibitors, and HDL levels were increased, which is good cholesterol. Most adverse cardiac events (MACE) were reduced after the use of PCSK9 inhibitors.

**Conclusion:**

In conclusion, ezetimibe, a PCSK9 inhibitor added to statin therapy, further reduces MACE risk without affecting all-cause mortality, even though statins already significantly reduce major adverse cardiovascular events (MACE) and mortality.

## 1 INTRODUCTION

Reducing the risk of atherosclerotic cardiovascular disease is the aim of lipid-lowering therapy (ASCVD). It is commonly acknowledged that low-density lipoprotein (LDL) is a major cause of ASCVD [1-3]. The first pharmacological option for lowering plasma levels of low-density lipoprotein (LDL-C) is to take statins [4, 5]. Statins have been demonstrated to significantly reduce the risk of ASCVD in both primary and secondary prevention settings by several outcome trials [6]. Guidelines for managing blood cholesterol recommend statin therapy for several patient groups. Primary prevention patients without diabetes but with at least a 7.5% risk of cardiovascular disease over the next decade are included. Those receiving secondary prevention who do not have diabetes also benefit. Familial hypercholesterolemia patients require treatment. Additionally, patients aged 40-75 with diabetes and LDL cholesterol over 70 milligrams per deciliter should use statins. The current standards aim to help high-risk individuals through medication while balancing disease severity and risk factors [7, 8]. Nevertheless, a significant residual risk of atherosclerotic cardiovascular disease (ASCVD) remains even with the best statin treatment [7-10]. Therefore, the development of novel drugs with the ability to efficiently reduce plasma LDL-C levels and other atherogenic particles is clinically necessary.

A new magnificence of medicine known as PCSK9 inhibitors is meant to lessen low-density lipoprotein (LDL), or “bad” cholesterol. These inhibitors have the potential to revolutionize the management of atherosclerotic risk. A liver-produced enzyme referred to as proprotein convertase subtilisin/kexin type nine (PCSK9) attaches itself to the low-density lipoprotein receptor (LDLR) of hepatocytes. The degradation of LDLR due to this binding increases the degrees of LDL-C inside the plasma [11-15]. The FDA has authorized evolocumab (Repatha) and alirocumab (Praluent). Their development represents a primary step forward in the field of LDL cholesterol reduction therapy, particularly for individuals with an increased risk of atherosclerotic cardiovascular disease [2, 16-21]. A gene on chromosome 1 encodes PCSK9, which was identified by researcher Nabil Seidah from Montreal, Canada. Through collaboration with a French research institution, Seidah and his team discovered that a gain-of-function mutation in this gene led to familial hypercholesterolemia in a French family [22]. Subsequent findings from investigators in Oslo, Norway, underscore the function of PCSK9 in lipid metabolism and its effects on cardiovascular well-being [23]. On the other hand, a report from the Dallas Heart Study showed that amongst a subgroup of African Americans, loss-of-function mutations in the PCSK9 gene have been associated with notably lower cholesterol levels and a markedly decreased incidence of cardiovascular disorder (CVD). These findings indicate the crucial role of PCSK9 in lipid metabolism and its potential association with variations in cardiovascular health across different populations [4, 24-29].

The baseline cardiovascular risk in an individual determines the absolute cardiovascular benefits of therapies [30, 31]. To date, however, no significant trial or meta-analysis has evaluated the possible absolute incremental effects of PCSK9 inhibitors and ezetimibe, either alone or in combination, by quantifying the degree of absolute cardiovascular risk reductions with these treatments in patients who are statin-intolerant or using maximally tolerated statins across various cardiovascular risk groups [32-40].

### 1.1 Rationale

Additional lipid-lowering strategies are necessary because atherosclerotic cardiovascular disease (ASCVD) continues to be a burden even with optimal statin therapy. Despite their effectiveness, statins do not completely reduce low-density lipoprotein cholesterol (LDL-C) and other atherogenic particles, which leaves a residual risk of ASCVD. Newer lipid-lowering medications like ezetimibe and PCSK9 inhibitors have demonstrated promise in further lowering LDL-C levels. By inhibiting the LDL receptors of hepatocytes from degrading, PCSK9 inhibitors improve the clearance of LDL-C, and ezetimibe, in addition to statins, prevents the small intestine from absorbing cholesterol. Nevertheless, it is unclear how exactly these drugs will benefit the cardiovascular system, either alone or in combination, especially when used with maximally tolerated statins or in patients who are intolerant to them. Furthermore, it is unclear what the additional benefits are for various baseline cardiovascular risk groups.

### 1.2 Objectives

The objective of this meta-evaluation and systematic assessment is to evaluate the effectiveness of recent lipid-decreasing medicines, especially ezetimibe and PCSK9 inhibitors, in reducing low-density lipoprotein cholesterol (LDL-C) in patients with atherosclerotic cardiovascular disease (ASCVD) who are either statin-intolerant or on maximally tolerated statin remedy. This study additionally aims to evaluate the extent to which these medications reduce the risk of major cardiovascular events, including stroke, myocardial infarction, and cardiovascular mortality. It examines the cardiovascular advantages of ezetimibe and PCSK9 inhibitors with regard to diverse baseline cardiovascular risk groups. Along with evaluating the safety profiles and tolerability of these agents, this review also identifies any adverse effects and their impact on patient adherence (Table **1**).

## 2 METHODOLOGY

### 2.1 Study Design

The following is the summary of the study design.

### 2.2 Eligibility Criteria

The following are the inclusion criteria for this study:

1. Adults (≥18 years) with atherosclerotic cardiovascular disease (ASCVD), heterozygous familial hypercholesterolemia, or clinical ASCVD-like disorder who have a high risk for heart attack/other acute events.
2. PCSK9 inhibitors (alirocumab and evolocumab) or ezetimibe when used as a monotherapy, with statins alone.
3. Placebo, no intervention, or alternative lipid-regulatory drugs.
4. References in scientific journals.
5. Methods: Publications in the English language that meet stress-related adaptation associated with an increased risk for cardiovascular disease guidelines, published within the past 5 years.

The exclusion criteria are as follows: 1. Studies concerning pediatric populations (under 18 years) or the ones without a clear prognosis of ASCVD or high cardiovascular risk. 2. Studies that do not include PCSK9 inhibitors as a part of the remedy routine. 3. Studies that do not report on cardiovascular outcomes, lipid levels, or safety profiles. 4. Research published in languages other than English because there are not enough resources for translation. 5. Overlapping datasets from the same research population or duplicate publications.

### 2.3 Search Strategy

PRISMA guidelines were adhered to during the article search process. There were various full-text articles, abstracts, and journal titles. To further refine the article search, more filters were suggested (Table **2**).

### 2.4 Selection Process

We searched peer-reviewed journals and publications on relevant literature. Studies were included or excluded based on specific inclusion and exclusion criteria. Exclusion criteria were as follows: (1) the population was not appropriate; (2) there was a significant risk of bias; (3) the study measured outcomes incorrectly; or (4) the study's design was inadequate for the purposes of our analysis. In some cases, multiple exclusionary factors combined, leading to a compounded effect.

### 2.5 Statistical Analysis

The statistics for each variable were manually extracted to conduct a meta-analysis. For dichotomous variables, the total sample size and event occurrences were calculated for both experimental and control groups. Additionally, whilst data were presented graphically, which consisted of figures, efforts were made to estimate numerical values for the findings. The primary analysis included trials that yielded pertinent outcomes. Heterogeneity, indicating true variation in effect sizes, was assessed using a threshold of *P <* 0.1. Heterogeneity was used to assess the extent of disparity amongst research studies, with a 50% difference considered substantial.

### 2.6 Quality Assessment

The Cochrane Risk-of-Bias tool was used to evaluate the quality of the studies, and a traffic light plot was drawn [41, 42].

## 3 RESULTS

### 3.1 Data Items

Fig. (**1**) and Table **3** show the PRISMA flowchart and summary of the included studies [43].

### 3.2 Study Characteristics

This systematic review included ine randomized controlled trials (RCTs) published between 2021 and 2024. The studies demonstrate geographical diversity, 37 being from Australia, China, anada, South Africa, the United States, Switzerland, etc. Sample size ranged from small cohorts (n=56–161) o large trials (n=18,924), with a total population of 22,247 participants.

The populations enrolled in the studies ere mostly high cardiovascular morbidity patients with ASCVD, ACS, HeFH/HoFH, and PCI. Inhibitors of proprotein convertase subtilisin/kexin (PCSK9), namely evolocumab, alirocumab, and inclisiran, utilized either s monotherapy or associated with statins Comparators were placebo, sham ontrols, or standard lipid-lowering therapy.

Main outcomes measured were LDL-C lowering, changes in total cholesterol, high-density ipoprotein (HDL), triglycerides, and MACE (myocardial infarction, stroke, and cardiovascular mortality). Imaging-based studies assessed tructural outcomes (*e.g.,* coronary plaque regression [fibrous cap thickness, lipid arc]).

Significant reductions in LDL-C were found with PCSK9 inhibitors compared with controls, with evolocumab and alirocumab showing LDL-C reductions of up o 72.9%. MACE outcomes, however, were heterogeneous with tatistically significant reductions in the risk reported in some studies but neutral effects in other studies, especially in target LDL-C populations. Overall there was geographical and methodological heterogeneity, with follow-up durations ranging from 45 days to 96 weeks, nd using different outcome measurement tools. These features illuminate the heterogeneity of the evidence base and situate the results of he meta-analysis.

### 3.3 Meta-Analysis

The meta-analysis was carried out using the Cochrane Collaboration's REVMAN software program, model 5.4.

### 3.4 Total Cholesterol

**Fig. (2):**
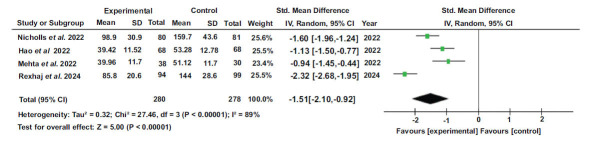
Forest plot of total cholesterol levels [44-46, 51].

**Fig. (3):**
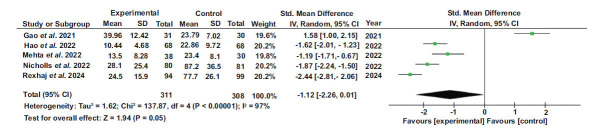
Forest plot of LDL levels [44-46, 50, 51].

**Fig. (4):**
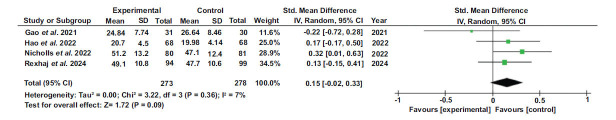
Forest plot of HDL levels [44, 45, 50, 51].

**Fig. (5):**
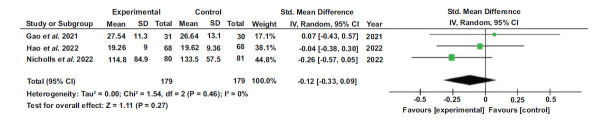
Forest plot of triglycerides levels [44, 45, 50].

**Fig. (6):**
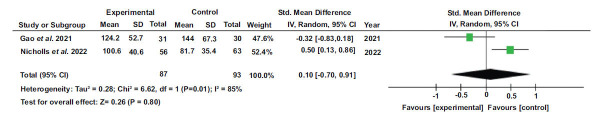
Forest plot of minimum fibrous cap thickness [44, 50].

**Fig. (7):**
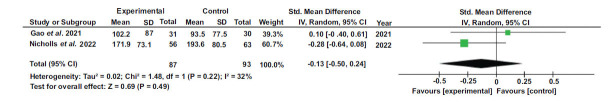
Forest plot of maximum lipid arc [44, 50].

**Fig. (8):**
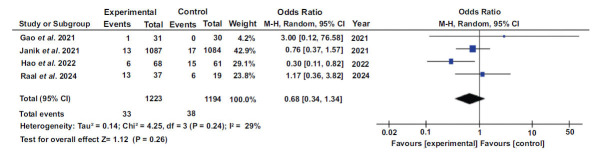
Forest plot of MACE: a graphical representation of the results from multiple studies that allows for comparing the effect of PCSK9 inhibitors on MACE across different studies [44, 46, 48, 51].

**Fig. (9):**
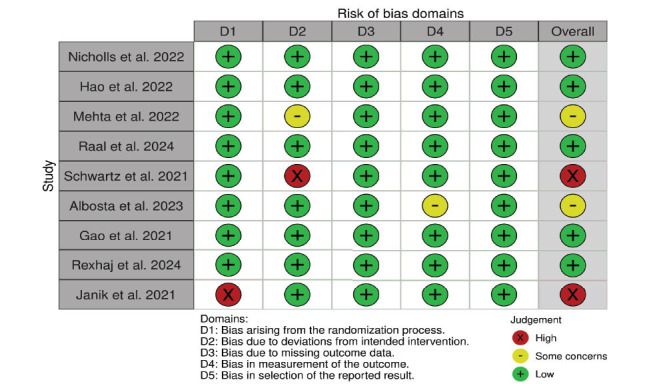
Traffic light plot of individual studies, which visually represents the risk of bias in each study. Green indicates low risk, yellow indicates some concerns, and red indicates high risk.

The overall effect size was d= -1.51, with a 95% confidence interval (-2.10, -0.92). The results indicated significant heterogeneity with I^2^=89%, df=3 (*p-*value<0.00001), tau^2^ = 0.32, and Chi^2^ = 27.46. The Z value was 5.00 (*p*<0.00001), indicating a significant overall effect. The results of each study separately favored the experimental group (Figs. **2**-**9**).

### 3.5 LDL Levels

The overall effect size was d=-1.12, with a 95% confidence interval (-2.26, 0.01). The results indicated significant heterogeneity with I^2^=97%, df=4 (*p*-value<0.00001), tau^2^ = 1.62, and Chi^2^ = 137.87. The Z value was 1.94 (*p*=0.05), indicating a significant overall effect. The results of each study separately favored the experimental group.

### 3.6 HDL Levels

The overall effect size was d=0.15, with a 95% confidence interval (-0.02, 0.33). The results showed heterogeneity with I^2^=7%, df=3 (*p*-value=0.36), tau^2^ = 0.00, and Chi^2^ = 3.22. The Z value was 1.72 (*p*=0.09), indicating a significant overall effect. The results of each study separately favored the control group.

### 3.7 Triglycerides Levels

The overall effect size was d=-0.12, with a 95% confidence interval (-0.33, 0.09). The results indicated the heterogeneity with I^2^=0%, df=2 (*p*-value=0.46), tau^2^ = 0.00, and Chi^2^ = 1.54. The Z value was 1.11 (*p*=0.27), indicating an overall effect. The results of each study separately favored the control group.

### 3.8 Minimum Fibrous Cap Thickness

The overall effect size was d=0.10, with a 95% confidence interval (-0.70, 0.91). The results indicated the heterogeneity with I^2^=85%, df=1 (*p*-value=0.01), tau^2^ = 0.28, and Chi^2^ = 6.62. The Z value was 0.26 (*p*=0.80), indicating the overall effect after analysis.

### 3.9 Maximum Lipid Arc

The overall effect size was d=-0.13, with a 95% confidence interval (-0.50, 0.24). The results demonstrated the heterogeneity with I^2^=32%, df=1 (*p*-value=0.22), tau^2^ = 0.02, and Chi^2^ = 1.48. The Z value was 0.69 (*p*=0.49), indicating the overall effect after analysis.

### 3.10 MACE

For four different studies, dichotomous data was plotted using the forest plot method. A random effects model was selected. The result was d=0.68, with a 95% confidence interval (0.34, 1.34). For the heterogeneity, the following values were determined: I^2^=29%, tau^2^=0.14, Chi^2^=4.25, and df=8 (*p*-value= 0.24). The Z value for the overall effect was found to be 1.12 (*p*=0.26). The results of each study separately favored the experimental group. The forest plot is shown in Fig. (**8**).

### 3.11 Risk of Bias Assessment

The ROBv2 was used in the study to assess and display the bias risk in 9 selected studies, as shown in Fig. (**9**).

## 4 DISCUSSION

A total of 9 studies were included in this systematic review and meta-analysis. Meta-analysis of the included studies showed that total cholesterol, LDL, and triglyceride levels were reduced after using PCSK9 inhibitors, and HDL levels were increased, which is good cholesterol. Most Adverse Cardiac Events (MACE) were reduced after the use of PCSK9 inhibitors. Following a non-ST-segment elevation myocardial infarction, statin therapy plus evolocumab produces beneficial modifications in coronary atherosclerosis, suggesting stabilization and regression. This raises the possibility of a reason behind the improved scientific results related to deficient LDL-C levels following acute coronary syndrome [44]. For patients at a high risk of acute coronary syndrome (ACS) with increased LDL-C levels, early integration of evolocumab into their treatment regimen may enhance lipid reduction, improve short-term LDL-C compliance, and yield better cardiovascular outcomes without increasing adverse reactions [45]. Alirocumab decreased LDL cholesterol by 22% compared to sham treatment in patients with a background of high-intensity statin therapy. However, a large-scale trial is required to decide if this simplified approach, accompanied by long-term remedy, improves cardiovascular results in patients with acute STEMI [46]. In patients who have recently experienced acute coronary syndromes and maintain LDL-C levels near 70 mg/dL despite optimized statin therapy, proprotein subtilisin/kexin type nine (PCSK9) inhibition provides additional clinical benefits only if their lipoprotein (a) concentration is at least mildly elevated [48]. Inclisiran, a novel siRNA therapy that reduces PCSK9 production, represents a valuable adjunct to maximally tolerated statin therapy ± ezetimibe for patients with clinical atherosclerotic cardiovascular disorder (ASCVD) or heterozygous familial hypercholesterolemia (HeFH) who require further LDL-lowering. Off-label, inclisiran may also be a promising choice for patients experiencing statin-associated side effects due to its precise mechanism of targeted uptake *via* hepatocytes [49].

Adding alirocumab to statin therapy now reduces LDL cholesterol and probably promotes a more stable plaque phenotype [50]. After 96 weeks of treatment, alirocumab showed no adverse effects on neurocognitive function, which was consistent with earlier findings on PCSK9 inhibitors. When used in patients with heterozygous familial hypercholesterolemia (HeFH) or those at high or very high cardiovascular risk, it significantly reduced LDL-C levels and was generally well tolerated [52].

In adults at very high or high cardiovascular risk who are receiving maximally tolerated statin therapy or who are statin-intolerant, ezetimibe or PCSK9 inhibitors may lower the incidence of non-fatal MI and stroke; however, this effect is not seen in those with moderate or low cardiovascular risk [2, 53-58]. The outcomes of this meta-analysis suggest that alirocumab may provide the greatest benefits in terms of all-cause mortality, with relatively lower risks of serious adverse events (SAEs), as both PCSK9 monoclonal antibodies and their inclusion enable patients to achieve the recommended LDL-C targets. Furthermore, for secondary prevention in patients at high risk of cardiovascular events, evolocumab can also offer the best benefits in reducing myocardial infarction. Future recommendations for the management of these patients should be informed by additional head-to-head trials with longer follow-up periods and rigorous methodological quality [24, 26, 59-68]. The strongest evidence for preventing cardiovascular complications comes from statins, which have been shown to lessen relative hazard by 10% to 15% for mortality and by approximately 25% for major adverse cardiovascular events (MACE) [6, 69-72]. Supplementing a statin regimen with ezetimibe, a PCSK9 inhibitor, or eicosapentaenoic acid ethyl ester also reduces the risk of MACE. However, it does not impact all-motive mortality [59, 69-81].

Our study had some limitations. Initially, our analysis focused on PCSK9 inhibitor medications, excluding comparisons with other classes, which may limit the generalizability of our results. Furthermore, significant heterogeneity was observed in the selected studies despite the lack of strong evidence for publication bias and the other outcomes reported from various guidelines. This significant heterogeneity may result from differences in the treatment protocols used in each of the included studies. It is important for future research to consider these limitations and design studies that can address them.

## CONCLUSION

Ultimately, while statins continue to be the cornerstone of cardiovascular risk reduction, consistently demonstrating significant reductions in MACE, the potential of PCSK9 inhibitors to further decrease the risk of major adverse cardiovascular events is a reason for hope. However, it is important to note that these additional treatment options do not impact all-cause mortality. The findings underscore the importance of optimizing lipid-lowering therapy strategies, particularly for high-risk patients, to maximize cardiovascular benefits while acknowledging the limitations in mortality outcomes. Future research should focus on long-term, high-quality head-to-head trials to refine and improve recommendations for managing patients with increased cardiovascular risks.

## Figures and Tables

**Fig. (1):**
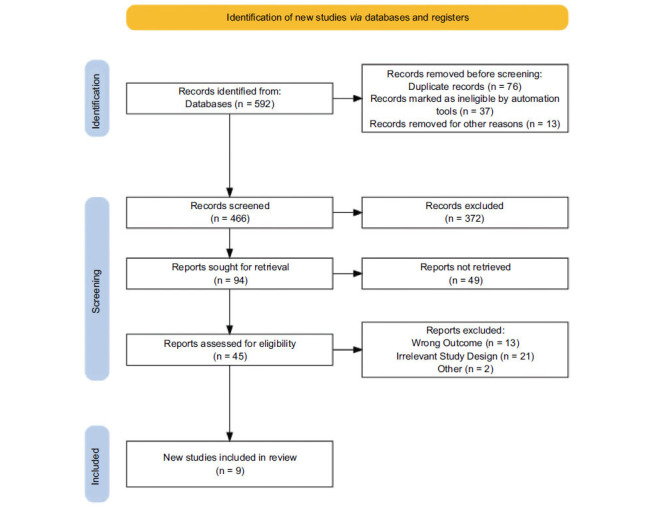
PRISMA flowchart of the included studies.

**Table 1:** PICO framework.

Population (P)	Individuals with atherosclerotic cardiovascular disease (ASCVD) or heterozygous familial hypercholesterolemia (HeFH) and those at high cardiovascular risk, who have LDL cholesterol levels that remain inadequately managed and the most common candidates for this medication. A total of 22,247 patients were included in the studies.
Intervention (I)	Treatment with PCSK9 inhibitors (alirocumab, evolocumab) or ezetimibe, either as monotherapy or in combination with statins.
Comparison (C)	Placebo, no treatment, or other lipid-lowering therapies.
Outcomes (O)	Low-density lipoprotein cholesterol (LDL-C) levels decrease the frequency of composite MACE, including myocardial infarction, stroke, and cardiovascular death, and changes in other lipid variables like triglycerides, HDL-C, and total cholesterol.

**Table 2:** Search strategy for the SRMA.

**Database**	**Search String**	**Number of Hits**
PubMed	(“PCSK9 inhibitors” [MeSH Terms] OR “Proprotein Convertase 9” [MeSH Terms] OR “Bempedoic Acid” [MeSH Terms] OR “Inclisiran” [MeSH Terms] OR “novel lipid-lowering agents” [All Fields] OR “new lipid-lowering drugs” [All Fields] OR “cholesterol-lowering agents” [All Fields] AND (“cardiovascular risk” [All Fields] OR “cardiovascular events” [All Fields] OR “cardiovascular disease” [MeSH Terms] OR “myocardial infarction” [MeSH Terms] OR “stroke” [MeSH Terms] OR “major adverse cardiovascular events” [All Fields] OR “MACE”[All Fields]).	565
Cochrane Library	“Atherosclerotic cardiovascular disease” or ASCVD or “heterozygous familial hypercholesterolemia” or HeFH or “adults aged 18 years or older with high cardiovascular risk” “PCSK9 inhibitors” or “proprotein convertase subtilisin/kexin type 9 inhibitors”, such as Alirocumab, Praluent, Evolocumab, or Repatha, or Ezetimibe, or Zetia Placebo or “no lipid-lowering treatment” or “other lipid-lowering therapies alone” “Reduction in low-density lipoprotein cholesterol” or “changes in total cholesterol, HDL-C, and triglycerides” or “occurrence of major cardiovascular events like myocardial infarction or stroke” or “cardiovascular mortality” or “safety and tolerability profile, including rates of adverse effects, leading to drug discontinuation” “Randomized placebo-controlled trial” or “prospective cohort study” or “case-control study” Adults aged 18 years or older with atherosclerotic cardiovascular disease, heterozygous familial hypercholesterolemia, or high cardiovascular risk receiving PCSK9 inhibitors as part of their lipid-lowering treatment plan evaluated in randomized controlled trials, cohort studies, or case-control studies for lowering of LDL-C and occurrence of major cardiovascular events as well as changes in other lipid parameters and safety.	27

**Table 3:** A summary of the included studies.

**Sr. No.**	**Study**	**Study Design**	**Location**	**Sample Size**	**Population**	**Intervention**	**Drug Used**	**Comparator**	**Outcomes**	**Main Findings**
1	Nicholls *et al*., (2022) [44]	RCT	Australia	161	Individuals over the age of eighteen who exhibited at least one non-culprit coronary artery blockage of 20% or more on angiography during a non-STEMI event were permitted to join the study.	Proprotein convertase subtilisin kexin type-9 (PCSK9) inhibitors	Evolocumab	Placebo	Total cholesterol levels, LDL, HDL, and triglycerides levels, minimum fibrous cap thickness, and maximum lipid arc	The group receiving evolocumab accomplished markedly lower LDL-C levels (28.1 *vs.* 87.2 mg/dL; *P <* 0.001). This treatment cluster also observed a more substantial growth in minimum fibrous cap thickness (+42.7 *vs.* +21.5 μm; *P =* 0.0.5), a greater reduction in maximum lipid arc (-57.5° *vs.* -31.4°; *P =* 0.04), and a more significant decrease in macrophage content (-3.17 *vs.* -1.45 μm; *P =* 0.04) throughout the arterial stage. Related beneficial effects were seen in lipid-rich plaque territories. Additionally, those given evolocumab experienced a more notable regression in percentage atheroma volume compared to the placebo group (-2.29% ± 0.47% *vs.* -0.61% ± 0.46%; *P =* 0.009).
2	Hao *et al*., (2022) [45]	RCT	China	136	Overall, 136 patients with high-risk acute coronary syndrome (ACS) and multiplied LDL-C tiers were included in the study.	Proprotein convertase subtilisin kexin type-9 (PCSK9) inhibitors	Evolocumab	Placebo	Total cholestrol levels, LDL, HDL, and triglycerides levels, MACE	While the average LDL-C level within the evolocumab group decreased substantially from 3.54 to 0.57 mmol/L over the course of one month, the control group experienced a more modest decline, from 3.52 to 1.26 mmol/L during that same period. Achieving an LDL-C threshold under 1.0 mmol/L was realized far more often among those administered evolocumab compared to the control group, with compliance rates of 82.35% *versus* 22.06%, respectively, a difference shown to be statistically significant at *P <* 0.01.
3	Mehta *et al*., (2022) [46]	RCT	Canada	68	68 patients with STEMI undergoing primary percutaneous coronary intervention (PCI).	Proprotein convertase subtilisin kexin type-9 (PCSK9) inhibitors	Alirocumab 150 mg subcutaneously	Sham-control group	Total cholesterol, LDL	At a mean of forty-five days, LDL cholesterol was reduced by 72.9% using alirocumab (from 2.97 mmol/L to 0.75 mmol/L) compared to a 48.1% decrease with the sham (from 2.87 mmol/L to least 1.30 mmol/L), ensuing in a mean between-group difference of -22.3% (*P <* 0.001).
4	Raal *et al*., (2024) [47]	RCT	South Africa	56	Provided subjects were above 18 years old with a genetic verification or clinical proof of homozygous familial hypercholesterolemia (HoFH) based on untreated LDL-C greater than 500 mg/dL from the history of hyperlipidemia.	Proprotein convertase subtilisin kexin type-9 (PCSK9) inhibitors	Inclisiran	Placebo	MACE	The average LDL-C concentrations at the beginning of the trial were 294.0 mg/dL in the inclisiran group and 356.7 mg/dL in the placebo group. On day 150, the change (expressed as a percentage) from baseline to each group of nondrug levels was 95% CI - 1.68 percent (−29.90% to 25.83% point) with *P =* 0.90. There was no statistical significance difference between the inclisiran group and placebo group in placebo-corrected changes on day 150.
5	Schwartz *et al*., (2021) [48]	RCT	USA	18,924	Patients with cardiovascular risk.	Proprotein convertase subtilisin kexin type-9 (PCSK9) inhibitors	Alirocumab	Placebo	-	In the group of patients with low LDL-C levels, those treated with a placebo (5.1 per 100 patient-years) had an incidence rate for major adverse cardiovascular events that was 4.2 per 100 patient-years higher than those with lower LDL-C levels (13.7 mg/dl). Rates of MACE for these patients on therapy were 3.1 per 100 people in one year, almost equal to rates observed in the crossover group (median). There was a very significant interaction between treatment and baseline lipoprotein (a), as indicated by a 95% confidence interval of the corresponding adjusted treatment hazard ratios of 0.68 (95% CI: 0.52–0.90) and 1.11 (95% CI 0.83–1.49) (*P*-value = 0. 017).
6	Albosta *et al*., (2023) [49]	RCT	USA	535	Patients with cardiovascular risk.	Proprotein convertase subtilisin kexin type-9 (PCSK9) inhibitors	Inclisiran	Placebo		For patients with heterozygous familial hypercholesterolemia (FH) or clinical atherosclerotic cardiovascular disease (ASCVD) who need further lowering of LDL cholesterol, inclisiran is currently FDA-approved as an adjuvant to statin therapy. This medication is injected subcutaneously twice a year, with an initial dose given three months apart.
7	Gao *et al*., (2021) [50]	RCT	China	61	Patients with cardiovascular risk.	Proprotein convertase subtilisin kexin type-9 (PCSK9) inhibitors	Alirocumab 75 mg Q2W	Standard care	LDL, HDL, triglycerides levels, minimum fibrous cap, maximum lipid arc	Cholesterol levels of LDL were significantly decreased in both the alirocumab and conventional care groups. Patients who received alirocumab had a much more marked reduction (1.72 ± 0.51 *vs.* 0.96 ± 0.59) compared with standard care only.
8	Rexhaj *et al*., (2024) [51]	RCT	Switzerland	139	Patients with cardiovascular risk.	Proprotein convertase subtilisin kexin type-9 (PCSK9) inhibitors	Alirocumab	Placebo	Total cholesterol, LDL, and HDL levels	Between the alirocumab group (n = 68, 5.44 ± 2.24%) and the placebo group (n = 71, 5.45 ± 2.19%) at 52 weeks, there was no difference in flow-mediated dilation (FMD) (difference = -0.21%, 95% CI -0.77 to 0.35, *P =* 0.47). In both groups, FMD improved similarly over the course of 52 weeks (*P <* 0.001).
9	Janik *et al*., (2021) [52]	RCT	USA	2176	Men and women between the ages of 40 and 85 who were at high or very high cardiovascular risk but did not have familial hypercholesterolemia (non-FH) or heterozygous for the condition were included in the study.	Proprotein convertase subtilisin kexin type-9 (PCSK9) inhibitors	Subcutaneous alirocumab 75/ 150 mg	Placebo	MACE	Over the 96-week period, exploratory endpoints for neurocognitive function in CANTAB domains did not reveal any significant differences between treatment groups or marginal inferiority. Alirocumab caused a nominal lowering of LDL-C and other lipids. Allergic reactions during dosing were rare.

## Data Availability

All the data supporting the findings of the study are provided within the article.

## LIST OF ABBREVIATIONS

ACS: Acute Coronary Syndrome
CVD: Cardiovascular Disorder
HeFH: Heterozygous Familial Hypercholesterolemia
LDL: Low-density Lipoprotein
LDL-C: Low-density Lipoprotein Cholesterol
LDLR: Low-density Lipoprotein Receptor
MACE: Most Adverse Cardiac Events
PCSK9: Proprotein Convertase Subtilisin/Kexin Type Nine
SAEs: Serious Adverse Events

## STANDARDS OF REPORTING

PRISMA guidelines and methodologies were followed.

## SUPPLEMENTARY MATERIAL

PRISMA checklist is available as supplementary material on the publisher’s website along with the published article.
